# Supplementary material for: Cross hybridization inference for phylogenetic resolution-fluorescence in situ hybridization enables microbiome imaging with strain level taxonomic resolution
Source: ISME Commun. 2026 Oct 13;6(1):ycag296. doi: 10.1093/ismeco/ycag296 (PMC13730059; doi:10.1093/ismeco/ycag296)
Supplement: Supplementary file 1 [file supplementaryrev1_ycag296.pdf]

Supplementary information for:

Cross hybridization Inference for Phylogenetic Resolution (CIPHR)-FISH enables microbiome imaging with strain level taxonomic resolution

Emmanuel E. Adade[1,2], Ruogu Wang[1,2], Colin M. Hennebery[1], Alex A. Lemus[1], Rebecca J. Stevick[3], David P. Pascual[3], Bianca Audrain[3], Alexa Orsino[2], Dylan Farnsworth[2], Jean-Marc Ghigo[3], Alex M. Valm[1,2,]

Supplementary Tables

Table S1. Salient features of bacterial strains and encoding FISH probes used in this study.

**Bacteria strain (Code)**	**Probe name**	**Encoding robe sequences (5'- 3')**	**Vendor**
Aeromonas caviae (UGB3607)	Acav144	CCAACTGTTATCCCCCTC	IDT
Aeromonas veronii 1 (UGB3608)	Aver448	AACGTCATTCCTCCTCGC	IDT
Aeromonas veronii 2 (UGB3609)	Aver448	AACGTCATTCCTCCTCGC	IDT
Phyllobacterium myrsinacearum (UGB3611)	Pmys187	GCTCATCCATCTCCGATA	IDT
Chryseobacterium massiliae (UGB3610)	Cmas154	CTATCCACCGGAGTTTTC	IDT
Pseudomonas mossellii (UGB3612)	Pmos86	CGCCGCTGAATCAAGGA G	IDT
Pseudomonas nitroreducens (UGB3613)	Pnit855	GACTTATCGCGTTAGCTG	IDT
Pseudomonas peli (UGB3614)	Ppel319	CCAACTTACTGAACCAC C	IDT
Pseudomonas sediminis (UGB3615)	Psed137	CGGACGTTATCCCCCACT	IDT
Stenotrophomonas maltophilia (UGB3616)	Smal138	TCCCCCACGACAGAGTA G	IDT

Table S2. Encoding and Readout probe sequences and their conjugated fluorophores used in this study.

**Bacteria strain (Code)**	**Probe**	**Probe sequences (5'- 3') Encoding Sequence_linker_ReadoutSequence**	**Readout probes seq. (5'-3') (Shi et.al., 2020)**	**Fluoro-phores**	**Vendor**
Aeromonas caviae (UGB3607)	Acav 144	CCAACTGTTATCCCCCTCCTGTGTGGAG GGATTGAAGGATA	TATCCTTCAATCC CTCCACA	Alexa Fluor 488	IDT
Aeromonas veronii 1 (UGB3608)	Aver4 48	AACGTCATTCCTCCTCGCACTTGAAAGG AATGGGTTGTGGT	ACCACAACCCATT CCTTTCA	Alexa Fluor 514	IDT
Aeromonas veronii 2 (UGB3609)	Aver4 48	AACGTCATTCCTCCTCGCACTTGAAAGG AATGGGTTGTGGT	ACCACAACCCATT CCTTTCA	Alexa Fluor 514	IDT
Phyllobacterium myrsinacearum (UGB3611)	Pmys 187	GCTCATCCATCTCCGATATTAGATGATG TAGTAGTAAGGGT	ACCCTTACTACTA CATCATC	Alexa Fluor 610	IDT
Chryseobacterium massiliae (UGB3610)	Cmas 154	CTATCCACCGGAGTTTTCTTAAGGGTGT GTTTGTAAAGGGT	ACCCTTTACAAAC ACACCCT	Alexa Fluor 660	IDT
Pseudomonas mossellii (UGB3612)	Pmos 86	CGCCGCTGAATCAAGGAGCTTATAGGA AATGGTGGTAGTGT	ACACTACCACCAT TTCCTAT	Alexa Fluor 532	IDT
Pseudomonas nitroreducens (UGB3613)	Pnit8 55	GACTTATCGCGTTAGCTGGCGTAGAGTT GATAGAGGGAGAA	TTCTCCCTCTATC AACTCTA	Alexa Fluor 594	IDT
Pseudomonas peli (UGB3614)	Ppel3 19	CCAACTTACTGAACCACCATGAGGTTA GGTTGAGAATAGGA	TCCTATTCTCAAC CTAACCT	Alexa Fluor 555	IDT
Pseudomonas sediminis (UGB3615)	Psed1 37	CGGACGTTATCCCCCACTTGGTGTGATG GAAGTTAGAGGGT	ACCCTCTAACTTC CATCACA	Alexa Fluor 647	IDT
Stenotrophomonas maltophilia (UGB3616)	Smal 138	TCCCCCACGACAGAGTAGTAAAGAGTG AGTAGTAGTGGAGT	ACTCCACTACTAC TCACTCT	Rhodamine Red X	IDT

Supplementary Figures

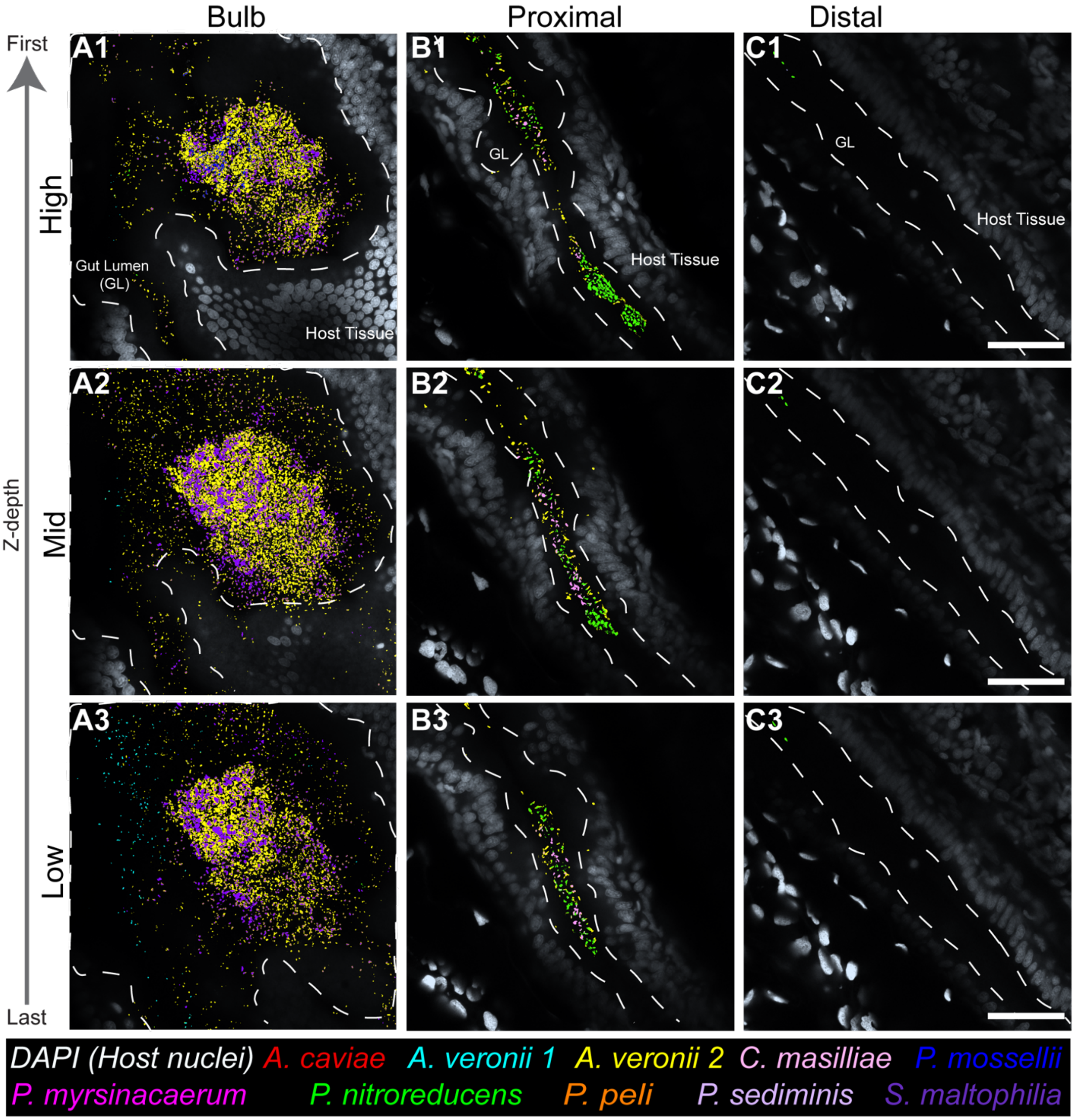

Figure S1. Species-level imaging of the larval zebrafish gut microbiome reveals spatially structured polymicrobial aggregates and internal variation across gut segments and through the axial depth of the gut in Fish 1. (**A1–A3**) Gut bulb biofilm architecture is dominated by *A. veronii*, with *S. maltophilia* and *P. mosselii* localized primarily within inner aggregate regions. (**B1–B3**) Proximal gut microbiome dominated by *P. nitroreducens* and *C. massilliae*. (**C1–C3)** Distal gut region predominantly colonized by *P. nitroreducens* with overall lower bacterial abundance. All panels show optical sections from confocal Z-stacks of species-resolved CIPHR-classified FISH signals from a defined 10-member microbial community. The dashed lines demarcate the gut epithelium boundary. Bar = 30 µm.

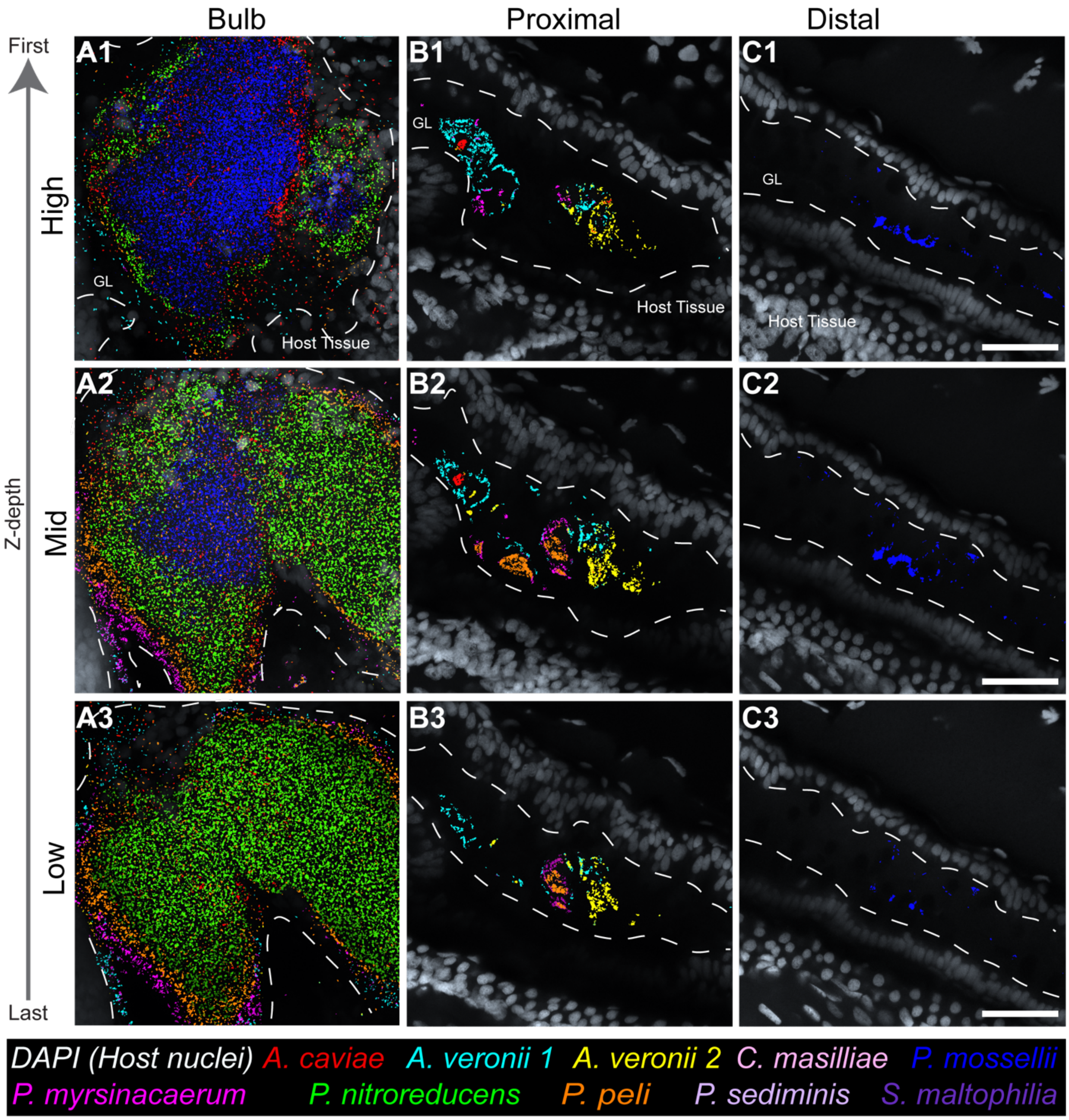

Figure S2. Species-level imaging of the larval zebrafish gut microbiome reveals spatially structured polymicrobial aggregates and internal variation across gut segments and along the Z-depth of Fish 3. (**A1–A3**) Gut bulb biofilm from a representative individual displays robust spatial organization. *Aeromonas veronii* (strains 1 and 2) and *A. caviae* localize near epithelial surfaces, while *P. myrsinacearum*, *P. peli*, and *P. mosselii* occupy outer aggregate regions. An inner core enriched in *Pseudomonas nitroreducens* and *P. mosselii*, with *A. caviae* distributed within the luminal biofilm space, is also observed (**B1–B3**) Proximal gut exhinited high microbial diversity with cells forming self-clustering micro aggregates (**C1–C3)** Distal gut region predominantly colonized by *P. mosselii,* with overall lower bacterial abundance. All panels show optical sections from confocal Z-stacks of species-resolved CIPHR-classified FISH signals from a defined 10-member microbial community. The dashed lines demarcate the gut epithelium boundary. Bar = 30 µm.

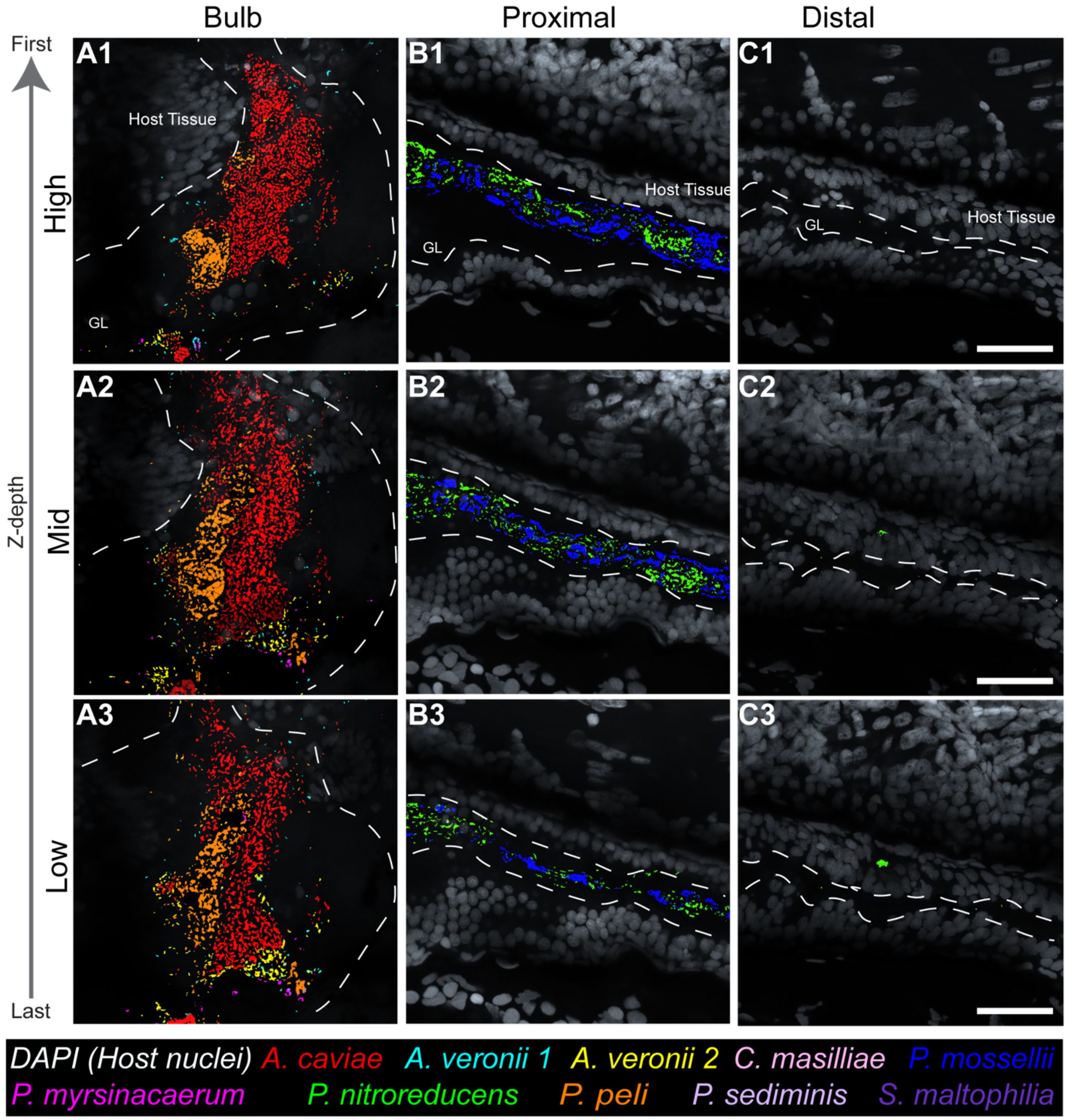

Figure S3. Species-level imaging of the larval zebrafish gut microbiome reveals spatially structured polymicrobial aggregates and internal variation across gut segments and along the Z-depth of Fish 2. (**A1–A3**) Gut bulb biofilm architecture dominated by *A. caviae*, with *P. peli* and *A.vaeronii* localized primarily within inner aggregate regions. (**B1–B3**) Proximal gut microbiome dominated by *P. nitroreducens* and *P. mossellli* in mono-species clusters (**C1–C3).** Distal gut region predominantly colonized by *P. nitroreducens,* with overall lower bacterial abundance. All panels show individual optical sections from confocal Z-stacks of species-resolved CIPHR-classified FISH signals from a defined 10-member microbial community. The dashed lines demarcate the gut epithelium boundary. Bar = 30 µm.

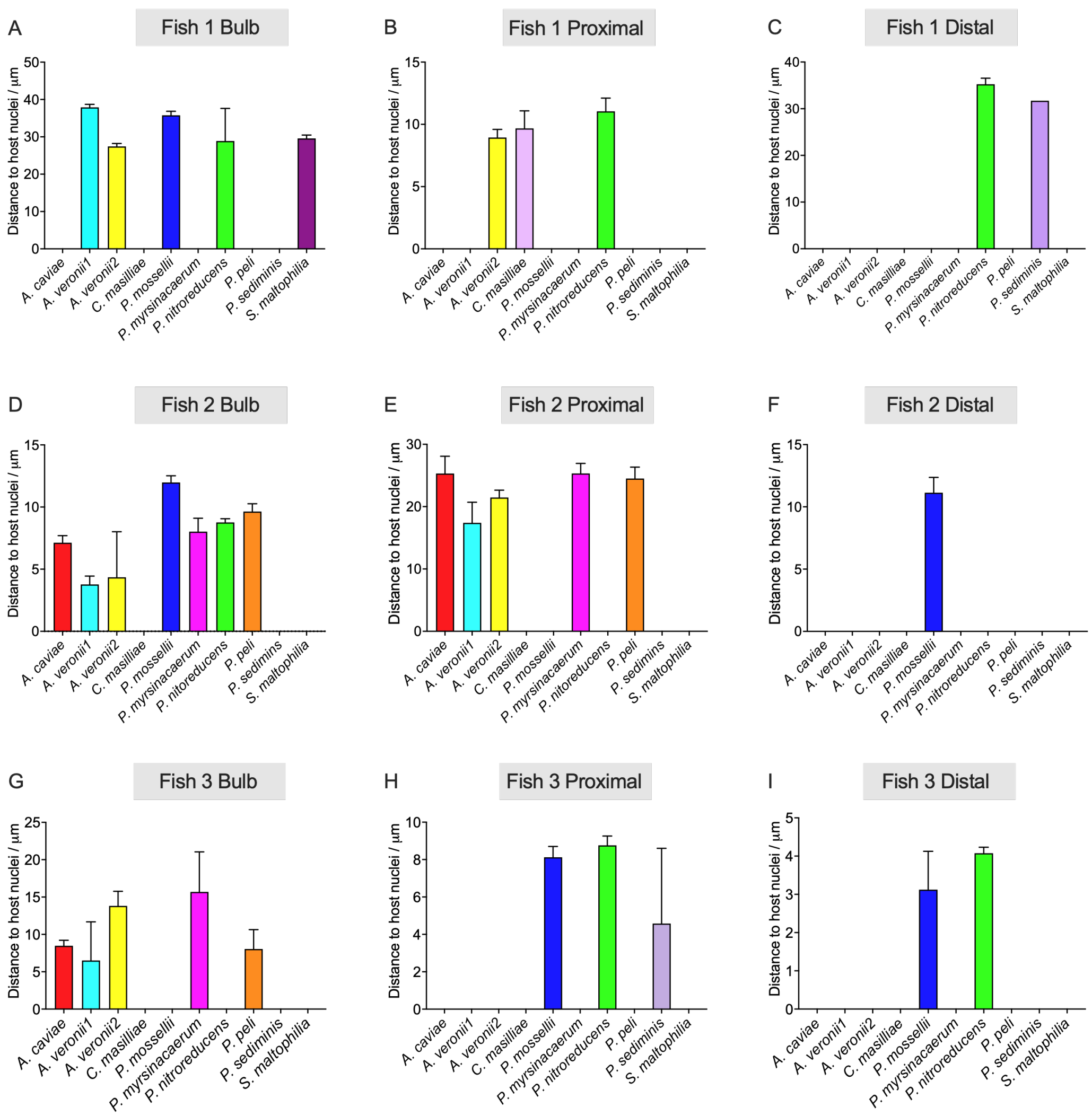

Figure S4. Spatial proximity of bacterial taxa to the host epithelial boundary. Median distances between cells of each bacterial species / strain and the epithelial boundary. Error bars represent the 95% confidence interval. Smaller distances indicate closer association with the host epithelium.

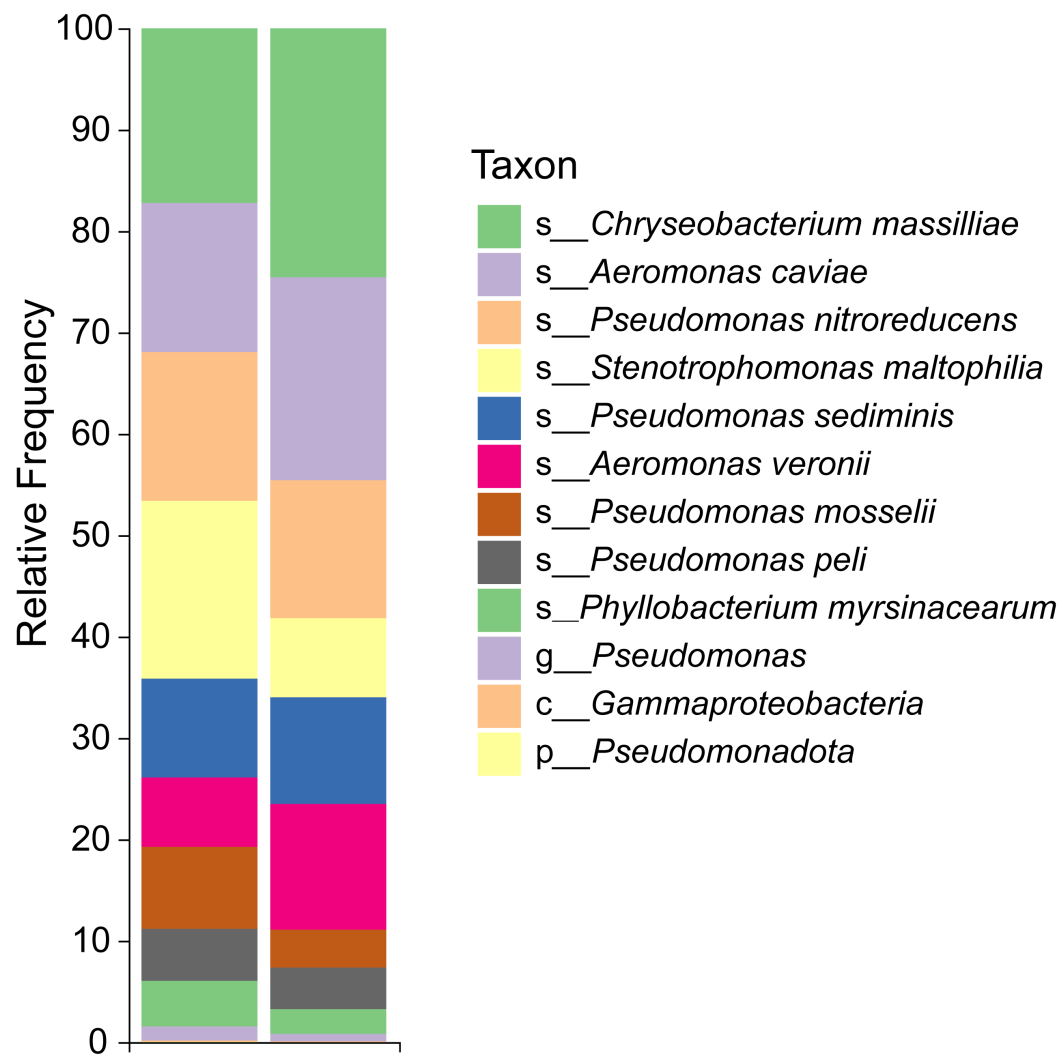

Figure S5. Full-length (V1–V9) 16S rRNA sequencing of reconventionalized zebrafish larvae colonized with a 10-member microbial consortium. DNA was extracted from whole zebrafish larvae in duplicate, with each replicate consisting of a pooled sample of four larvae to increase DNA yield.
